# Mitochondria-Targeted Antioxidant SS31 Prevents Amyloid Beta-Induced Mitochondrial Abnormalities and Synaptic Degeneration in Alzheimer’s Disease

**DOI:** 10.3390/ph5101103

**Published:** 2012-10-16

**Authors:** Marcus J. Calkins, Maria Manczak, P. Hemachandra Reddy

**Affiliations:** 1 Neurogenetics Laboratory, Division of Neuroscience, Oregon National Primate Research Center, Oregon Health & Science University, 505 NW 185th Avenue, Beaverton, OR 97006, USA; Email: manczakm@ohsu.edu (M.M.); reddyh@ohsu.edu (P.H.R.); 2 Center for Research on Occupational and Environmental Toxicology, Oregon Health & Science University, 3181 SW Sam Jackson Park Road, Portland, OR 97239, USA

**Keywords:** Alzheimer’s, SS31, mitochondria, antioxidant

## Abstract

In neuronal systems, the health and activity of mitochondria and synapses are tightly coupled. For this reason, it has been postulated that mitochondrial abnormalities may, at least in part, drive neurodegeneration in conditions such as Alzheimer’s disease (AD). Mounting evidence from multiple Alzheimer’s disease cell and mouse models and postmortem brains suggest that loss of mitochondrial integrity may be a key factor that mediates synaptic loss. Therefore, the prevention or rescue of mitochondrial dysfunction may help delay or altogether prevent AD-associated neurodegeneration. Since mitochondrial health is heavily dependent on antioxidant defenses, researchers have begun to explore the use of mitochondria-targeted antioxidants as therapeutic tools to prevent neurodegenerative diseases. This review will highlight advances made using a model mitochondria-targeted antioxidant peptide, SS31, as a potential treatment for AD.

## 1. Introduction

Alzheimer’s disease (AD) is the most common neurodegenerative disorder in aged populations worldwide. AD is characterized by deficits in cognition and memory, as well as personality and behavior [1]. Amyloid plaques and neurofibrillary tangles are the pathological hallmarks of AD [1,2], but since they both occur late in pathogenesis, they are unlikely to represent the primary cause of AD symptoms. Additional processes that have been identified in the etiology of AD include neuro-inflammation, synaptic damage, and structural and functional abnormalities in mitochondria [3,4,5,6,7,8,9,10]. Synaptic loss represents the best correlate to loss of memory and cognitive decline [11,12,13]. The mechanisms by which synaptic loss occur are largely unknown in AD, and the influence of early pathogenic events, such as mitochondrial dysfunction, on synaptic loss has not been fully described. The purpose of this article is to highlight the latest developments in AD research, regarding mitochondrial dysfunction and synaptic degeneration, with a special emphasis on the protective effects of the mitochondria-targeted antioxidant SS31 in AD neurons.

## 2. Alzheimer’s Disease Etiology Involves Mitochondrial Dysfunction

The initial cause of AD neurodegeneration is thought to be the abnormal processing of the amyloid beta (Aβ) precursor protein (AβPP), leading to Aβ formation. Evidence for this hypothesis comes from mutations that cause hereditary AD in humans and in animal models of AD [14]. Aβ is produced by the sequential cleavage of AβPP by β-secretase and γ-secretase yielding a 38–43 amino acid protein [2]. The most prevalent forms of Aβ are Aβ40 and Aβ42, of which Aβ42 is known to be more toxic. Aβ forms soluble aggregates (oligomers and protofibrils) as well as insoluble fibrils that comprise the amyloid plaques [15]. Current thought is that oligomeric Aβ is responsible for the initiation of synaptic loss in AD [14,16]. Aβ oligomers can accumulate in subcellular organelles and can induce synaptic degeneration and overt toxicity in neurons [17,18,19,20,21]. The role of mitochondria in oligomeric Aβ-induction of synaptic degeneration is currently an area of intense research [19,22,23,24,25,26].

Correlated changes in synaptic function and mitochondrial function occur as early events in AD [21,27]. Several lines of evidence demonstrate this point. First, Aβ is known to associate with mitochondrial membranes in its monomeric and oligomeric forms [28,29,30,31,32,33]. Additionally, Aβ interacts with mitochondrial proteins Drp1 [19], ABAD [34] and cyclophilin D [35]. Oligomeric Aβ is known to decrease ATP production and induce free radical production [36]. Finally, mitochondrial transport, fission and fusion are all disrupted by oligomeric Aβ [19,22,23,24,25,26]. It has therefore been proposed that oligomeric Aβ-induced mitochondrial deficiencies precede and contribute to synaptic degeneration and loss in AD [21,27,36].

## 3. Mitochondrial Function Is Tightly Coupled to Synaptic Activity

Mitochondria play several key roles in synaptic maintenance. The dynamic mitochondrial functions of ATP generation, Ca^2+^ regulation, and modulation of caspase activity are all essential for neuronal viability. In the case of ATP generation, it has long been recognized that neuronal activity can stimulate oxidative phosphorylation. One of the most thoroughly studied examples in this regard is the coupling of cytochrome c oxidase (COX) transcription to neural activity. Ca^2+^ regulation by synaptic mitochondria has also been carefully studied, and mitochondrial control of neurite pruning is an emerging field that has gained recent attention. For these three reasons, synaptic mitochondria are critical for the proper functioning and viability of synapses and neurons overall. Deficiencies in any of these areas would be expected to lead to drastic reductions in neuronal function.

Since neurons are non-dividing cells, the ATP that neurons consume is entirely utilized for cellular function, not replication. Therefore it has been highly informative to create energy budgets for neurons based on theoretical calculations. The major cellular process that hydrolyses ATP is the maintenance and re-establishment of the ion gradient, which is essential for neuronal activity. It is estimated that this process consumes approximately half of the ATP within neurons with another 30% dedicated to synaptic transmission [37,38]. The energy for these functions is used mainly in synapses and distal regions of the cell, and relies heavily on mitochondrial oxidative phosphorylation for ATP generation. It has been widely observed that neurons exhibit poor survival in anaerobic conditions, illustrating the previous point. Moreover, the preferred energetic substrate for the tricarboxylic acid (TCA) cycle in glutamatergic neurons is thought to be lactate, which is produced by anaerobic metabolism in astroctyes and is secreted by neurons for uptake [38,39,40]. It is well known that local energetic demand and production are strongly correlated with neuronal activity, and importantly it is neuronal activity that is responsible for enhanced metabolic activity, not vice-versa [41]. A clear demonstration of neural activity inducing ATP production comes from the upregulation of COX in active neurons. As early as 1989, Wong-Riley and colleagues demonstrated the use of COX activity as a surrogate marker for neuronal activity in the occipital cortex [41]. Since then, this method has gained wide acceptance. Subsequently, detailed molecular dissection of this phenomenon showed that COX regulation involves coordinated bigenomic transcription of both mitochondrial and nuclear-encoded genes [42]. In short, it appears that neuronal activity leads to activation of the nuclear respiratory factors 1 and 2 (Nrf-1 and Nrf-2) as well as the coactivator PGC1α [43]. These transcription factors operate coordinately to upregulate 13 nuclear genes encoding 10 *COX* subunits and three transcription factors (*Tfam*, *Tfb1m*, *and Tfb2m*) responsible for upregulation of the remaining three *COX* subunits within mtDNA [44]. Therefore, a picture of the mechanism by which stimulation of ATP production follows neuronal activity is emerging. Nrf-1, Nrf-2 and PGC1α not only regulate transcription of *COX*, but other mitochondrial constituents as well [43,44]. In fact, these proteins (especially PGC1α) are often regarded as master regulators of mitochondrial biogenesis [43,44,45,46,47], and as such, a likely conclusion is that neuronal activity stimulates mitochondrial biogenesis *per se*. It is still unknown what molecular signals recruit re-localization of the newly synthesized constituents and presumably an entire organelle to distal regions of the cell in order to satisfy the highly local energy demand for neural functions.

Mitochondrial Ca^2+^ clearance is also essential for correct synaptic function and overall neuronal survival. Ca^2+^ buffering is especially critical in the pre-synaptic and post-synaptic compartments where Ca^2+^ concentrations may vary widely and must be tightly regulated. Neuronal mitochondria are crucial for the central role they play in intracellular Ca^2+^ homeostasis due to their direct buffering capacity and their ability to generate ATP which fuels other mechanisms of Ca^2+^ mobilization [48]. Mitochondrial Ca^2+^ buffering is currently thought to account for a large proportion of the post-synaptic Ca^2+^ clearance after neuronal firing [49,50,51] and to be responsible for influencing Ca^2+^-dependent processes, such as synaptic vesicle release [52,53,54]. Using fluorescent probes Rhodamine123 (for mitochondria) and Fura-3 (for calcium), Kann and colleagues found the Ca^2+^ concentration of mitochondria to correlate with electrical activity in hippocampal slice cultures [49]. Further, Pivovarova *et al*. found that mitochondria quickly sequestered intracellular Ca^2+^ in CA3 neurons, but the endoplasmic reticulum responded more slowly in both uptake and release [51]. Therefore, the cooperative action of endoplasmic reticulum and mitochondria result in the finely tuned buffering of Ca^2+^ in neurons [55,56,57].

Recently, researchers found that neurite pruning is mediated largely by local caspase signaling [58,59,60,61,62] and that much of this signaling proceeds through mitochondrial control of the apoptotic machinery. The extrinsic pathway of apoptosis may contribute to neurite pruning during development [61]. However, the intrinsic pathway seems to be fundamentally required for the reduction in dendritic spines. This may provide the basis for long-term depression (LTD) of neurons [63,64,65] and the degeneration of synapses seen in AD. A role for caspase-3 in the formation of LTD has been suggested. Li *et al*. [66] used a combination of peptide inhibitors and knockout animals in hippocampal slice cultures to show that mitochondrial activation of caspase-3 and caspase-9 is necessary for the formation of LTD but not for long-term potentiation. They then extended this work to show that BAD and BAX proteins functioned upstream of caspase activation in LTD formation, in cultured hippocampal neurons and slices [67]. The involvement of mitochondria in AD-induced spine loss can be illustrated by focusing on AMPA-receptor degradation and coincident spine degeneration. AMPA-receptor surface expression was found to be decreased in cultures treated with Aβ, and this decrease was associated with spinal loss and decreased AMPA responsiveness [68,69]. Moreover, AβPP primary neurons exhibit decreased GluR1, AMPA receptor subunit, and expression [70]. D’Amelio and colleagues showed that in the Tg2576 mouse model of AD, mitochondria-mediated apaf-1 activation of caspase-3 leads to early loss of hippocampal dendritic spines, and that this activation also diminished post-synaptic AMPA receptors [60]. This neuritic simplification strongly correlated with behavioral abnormalities in AD mice that have been found long before the deposition of amyloid plaques [60]. In a study in which primary hippocampal neurons were exposed to Aβ oligomers, GluR1-immunopositive spines were rapidly lost (within 30 min) and mitochondria were implicated in protecting from the loss of AMPA receptors. The probability of an individual spine showing GluR1 immunoreactivity after treatment correlated strongly with the probability that a mitochondrion exists in close proximity to the spine [71].

Based on these studies, mitochondria appear to be central to proper regulation of AMPA receptor endocytosis and that a corresponding degeneration in spines and synapses is reliant on mitochondrial-mediated caspase activation [60,72]. Thus, the limited activation of caspases, which results in synaptic loss, may explain how behavioral deficits in AD may precede frank neuronal degeneration.

## 4. Mitochondria Are Highly Regulated in Neurons

**Figure 1:**
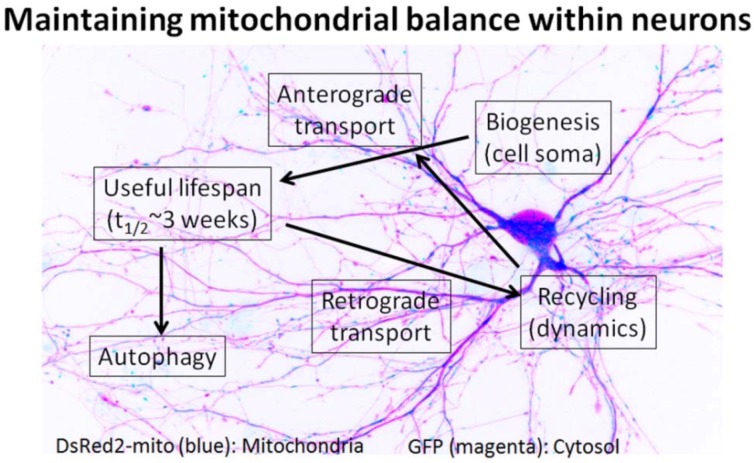
Primary hippocampal neuron with mitochondria labeled by MitoDsRed2 and cytosol labeled by GFP. The figure illustrates the mitochondrial lifecycle, including biogenesis, mitochondrial transport, and synaptic localization and degradation. Because of neuronal architecture with long processes and high energetic demands at distal regions of the cell, mitochondrial distribution is critical to the survival of neurons. Disruptions in regulating several aspects of mitochondrial biology are known to lead to neurodegeneration.

The mitochondrial lifecycle in neurons involves biosynthesis, distribution, recycling, and autophagy (Figure 1). Mitochondrial biosynthesis is thought to occur largely in the neuronal soma, based on pioneering work in which mitochondrial DNA (mtDNA) synthesis was studied in PC12 cells [73]. In this work, researchers reported that the incorporation of BrdU into mtDNA occurred in a perinuclear pattern, with incorporation decreasing the further the mitochondria were from the nucleus. In a subsequent study, however, the same researchers decoupled the synthesis of mtDNA from transcription of mitochondrial substituents. They found that the putative nuclear gene product required for mtDNA synthesis (*Polγ*) is transcribed in the absence of cellular mtDNA while the nuclear gene product for the mtDNA transcription factor *Tfam* is not [74]. More recently, researchers have found that protein synthesis occurs in the axons and dendrites of neurons [75,76,77], with mitochondrial proteins, in particular, being synthesized in axons [78,79].

Researchers also found that mtDNA replication occurs throughout the cell body of fibroblasts [80] and within axons that had been resected from the cell body [81]. In our own studies, we found that healthy neurons exhibited mtDNA replication mainly within the soma, but also to some extent within axons and dendrites [82]. Additionally, we found that a perinuclear localization of mitochondria was greater in neurons that had been treated with toxins, such as rotenone, hydrogen peroxide, and Aβ. Overall, it appears likely that mitochondrial biogenesis does, indeed, occur largely in the cell soma by virtue of the sheer abundance of protein synthesis machinery there, and to a lesser extent, it also occurs in axonal compartments.

**Figure 2:**
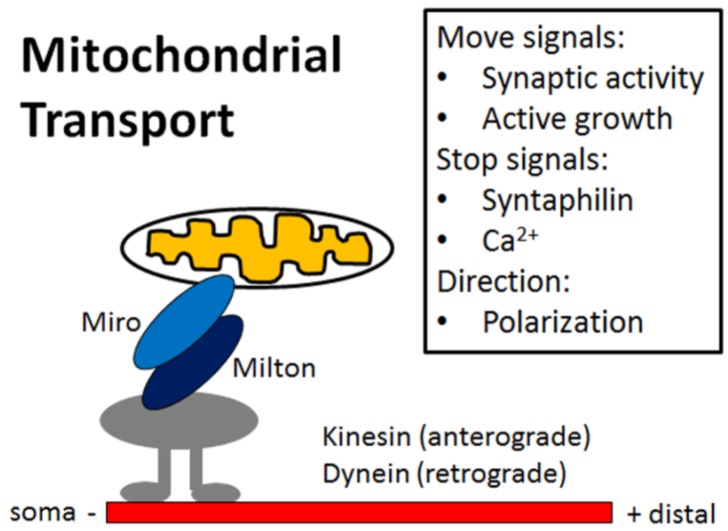
An illustration showing the transport of mitochondria from cell body to nerve terminal. Mitochondria are transported along microtubules and are attached to molecular motors by Miro and Milton. These two proteins provide important points of regulation for mitochondrial transport and may prove to mediate the signals for mitochondrial distribution.

Since newly synthesized mitochondria occur mainly in the cell soma, they must be transported to distal regions to function in ATP generation, Ca^2+^ buffering, and LTD formation. Mitochondrial transport processes are illustrated in Figure 2. Detailing the molecular mechanisms of mitochondrial transport, especially within neurons, is an important and intensive area of study. Currently, many of the key players have been identified in this complex and highly regulated process; however, the exact regulatory mechanisms and even many of the context-specific on/off signals are as yet undescribed [83]. Both synaptic activity and active growth are known to signal mitochondrial motility, while syntaphilin and Ca^2+^ are thought to provide stop signals. 

The direction of movement may be related to the polarization of the organelle; however, this has not been conclusively determined. Using the dye JC-1 to distinguish between polarized and depolarized mitochondria, Miller and Sheetz showed that polarized mitochondria tended to move anterograde, toward the distal synapses, while depolarized mitochondria tended to move retrograde [84]. However, in a more recent study using the mitochondrial dye TMRM, which may be a more reliable measure of mitochondrial potential, this pattern was not observed [85]. The transport machinery utilizes both kinesin and dynein motors, which operate along microtubules. Mitochondria are attached to the motors by two proteins miro and milton, which provide key points of regulation.

Decreased mitochondrial transport has been found in many diseases, including AD, amyotrophic lateral sclerosis (ALS), Huntington’s disease, and Parkinson’s disease [86,87,88]. In fact, in AD, current evidence suggests that this decrease in transport is an early event in neurodegeneration that precedes axonal loss [25,89,90,91]. Hydrogen peroxide treatment was shown to inhibit both mitochondria and Golgi-derived vesicle transport along axons demonstrating that oxidative stress can directly influence transport phenomena [92]. Potential mechanisms involved in the interference of mitochondrial transport include direct interference with transport machinery, alterations in move, stop or directional signals, or alterations in mitochondrial fission and fusion.

## 5. Impaired Mitochondrial Dynamics in Alzheimer’s Disease Neurons

**Figure 3:**
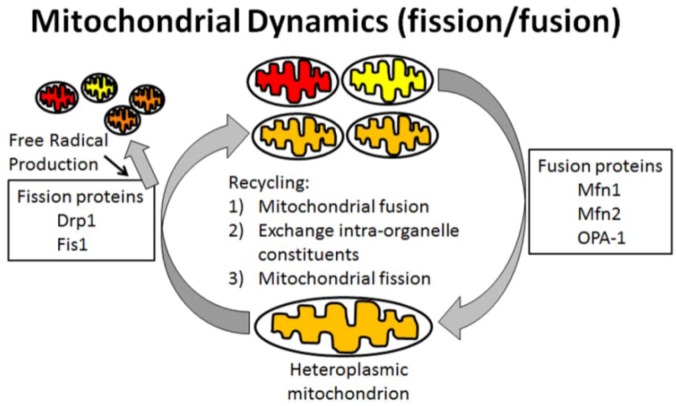
Illustration showing mitochondrial dynamics in a healthy neuron. Mitochondrial fission and fusion are the means by which mitochondria are recycled. Dilution of damaged mitochondrial constituents occurs by this process thereby extending the lifespan of otherwise non-functional organelles. Oxidative stress within the cell is known to result in mitochondrial fragmentation which disrupts normal mitochondrial cycling.

Mitochondrial dynamics is a process by which mitochondria divide and fuse in most eukaryotic cells. Mitochondrial shape and structure are maintained by equal and opposite forces: mitochondrial fission and mitochondrial fusion. In healthy cells, fission and fusion events balance equally, resulting in the maintenance of mitochondrial function. Mitochondria alter their shape and size to move from the cell body to the axons, dendrites, and synapses, and back to the cell body [36]. As described above, mitochondria are synthesized in the cell body and travel along the axons and dendrites to supply energy to nerve terminals for normal neural communication. After use, damaged mitochondria are thought to be recycled by fusion with healthy mitochondria, intraorganelle exchange of proteins, lipids and DNA and subsequent fission (Figure 3).

Fission and fusion are controlled by evolutionary conserved, dynamin related large GTPases. Fission is regulated by the dynamin-related protein (Drp1) and mitochondrial fission 1 (Fis1), of which the latter is localized to the mitochondrial outer membrane [36]. When a mitochondrion signals to divide, Drp1, a cytosolic protein, translocates to the mitochondrial outer membrane and to initiate the process of fragmentation.

Mitochondrial fusion is controlled by three GTPase proteins: two proteins located in the outer mitochondrial membrane mitofusins 1 [Mfn1] and 2 [Mfn2]), and one protein located in the inner membrane localized protein (optic atrophy 1 [Opa1]). The C-terminal portion of Mfn1 mediates oligomerization between Mfn molecules of adjacent mitochondria and facilitates mitochondrial fusion. 

Interestingly, direct links between mitochondrial fusion and transport are being uncovered in neurons. For example, Mfn2 was found to be required for mitochondrial transport [93]. Converesly, overexpression of the transport protein Miro was shown to increase mitochondrial fusion in cell lines [94,95].

Recent evidence from our lab [19,22,26] and others [23,24] suggests that impaired mitochondrial dynamics in AD neurons is present and furthermore may result from Aβ interaction with Drp1 and subsequent increased production of free radicals. In turn, this elevation of free radicals activates fission proteins Drp1 and Fis1 and cause excessive mitochondrial fragmentation, defective transport of mitochondria to synapses, low synaptic ATP and synaptic dysfunction in AD neurons [96,97].

## 6. Oxidative Stress in Mitochondria Is Associated with AD

The term *oxidative stress* is commonly offered to explain the balance between the production of oxidants and the endogenous antioxidant defenses in cells, including neurons. In general, cells undergo apoptotic death when there is an imbalance between endogenous antioxidant enzymes and oxidants produced within the mitochondria.

Mitochondria-linked oxidative stress has been found to be a major factor associated with the development and progression of AD [27]. Further, increasing evidence suggests that free radical associated oxidative damage of lipids, nucleic acids, and proteins is extensive in the brains of AD patients [27,97,98]. The brain appears to be more vulnerable to oxidative stress compared to other organs in the body. Oxidative stress markers, such as 8-hydroxyguanosine and hemeoxygenase, have been localized to pathologic sites in the brains of AD patients [98].

## 7. Mitochondria-Targeted Antioxidants May Be Beneficial in AD

Initial clinical application of antioxidants to prevent or delay symptoms of AD has met with limited success. This underwhelming response may be due at least in part to the non-specific nature of antioxidant therapies. Up to now, there are literally dozens of completed or ongoing clinical trials using such antioxidants as vitamin E, epigallocatechin gallate (EGCG), resveratrol, curcumin, pramipexole, latrepirdine, ubiquinone, lipoic acid, idebenone, *Ginkgo biloba*, and *N*-acetylcysteine [99]. Many of the non-traditional antioxidants are known to modulate glutathione levels in brain, and this strategy has been proposed as a potential treatment in AD [100]. Unfortunately, only a handful of these trials have yielded positive results, with several showing a negative association between antioxidant supplementation and positive outcomes. Because of challenges including blood brain barrier penetration, use of late stage AD patients, and other aspects of the trial design, it has been proposed that generalized antioxidants have limited potential compared to more targeted approaches [96].

Because oxidative damage in mitochondria is thought to be a driving force in the dysfunction of mitochondria, investigators have begun to examine the efficacy of antioxidant therapies specifically targeted to mitochondria. Several mitochondria-targeted antioxidants have been developed in this regard and are currently undergoing preclinical testing. Candidate molecules including MitoQ, MitoVitE, MitoPBN, MitoPeroxidase, glutathione choline esters, latripiridine and Szeto-Schiller (SS) peptides, have been developed and the potential use of these molecules to combat neurodegenerative disease has been previously detailed [27,97,101,102,103]. One of the most promising mitochondria-targeted antioxidants from this category is SS31 [27,96,97,98].

SS31 was developed as a part of a series of SS peptides that were synthesized and characterized as opioid agonists [104,105]. Soon thereafter, it was discovered that both SS02 (Dmt-D-Arg-Phe-Lys-NH_2_; Dmt represents 2',6'-dimethyltyrosine) and SS31 (D-Arg-Dmt-Lys-Phe-NH_2_) exhibited mitochondrial accumulation as well as *in vitro* inhibition of lipid peroxidation and H_2_O_2_ scavenging [106]. Furthermore, these mitochondria-targeted antioxidant molecules were found to protect from Ca^2+^-induced mitochondrial depolarization, the swelling and release of cytochrome c in isolated organelles, and ischemia reperfusion injury in guinea pig heart [106]. SS31 was then evaluated in models of islet cell transplantation [107], myocardial infarction [108], brain ischemia reperfusion [109], and ALS [110]. Strikingly, SS31 was found to be beneficial in all of these conditions.

Based on the neuroprotection capacity of SS31, the Reddy lab has evaluated SS31 in models of AD, including Aβ-treated N2a neuroblastoma cells, primary neurons from Tg2576 mice and aging Tg2576 mice. In N2a neuroblastoma cells treated with Aβ25-35, we observed a broad range of effects on mitochondria, suggesting dysfunction and increased oxidative stress [26]. First, mitochondrial fission genes and proteins were found to be upregulated, while fusion genes and proteins were down-regulated. There was also an accumulation of small, apparently fragmented, mitochondria within the cell body. Furthermore, Aβ treatment was associated with increased H_2_O_2_ production, along with decreased COX activity and ATP. Treating N2a cells with SS31in combination with Aβ provided at least partial rescue in all of these parameters, compared to Aβ alone. SS31 treatment alone was associated with increased expression of mitochondrial fission and fusion genes and proteins, decreased H_2_O_2_ production, increased COX activity, and increased ATP levels. These findings suggest that a rescue effect may be conferred by SS31. Our lab also evaluated the efficacy of SS31 in primary neurons from Tg2576 mice. These neurons are known to be deficient in synaptic markers and to exhibit dendritic simplification due to excreted Aβ and intracellular accumulation of oligomeric Aβ [111,112,113]. We found that in addition to synaptic loss, aged Tg2576 neurons have increased numbers of rounded somatic mitochondria and concurrent loss of dendritic mitochondria, reminiscent of cells that have been treated with Aβ [22]. This mitochondrial alteration is coincident with an intracellular accumulation of oligomeric Aβ [22] as well as dendritic simplification [26], and precedes overt neuronal degeneration. These processes were further associated with increased mitochondrial fission, as evidenced by gene expression and protein levels of mitochondrial dynamics genes, as well a pronounced decrease in anterograde mitochondrial trafficking. Treatment with SS31 was able to reverse both the trafficking deficit and the occurrence of excess mitochondrial fission.

Since trafficking and dynamics are highly interrelated processes, it is not surprising that we found coincident reversal of both endpoints with the addition of SS31. If we are to accept the hypothesis that mitochondrial health is a determinant for anterograde transport (as suggested in [84]), then the mechanistic link between antioxidant therapy and correct mitochondrial distribution would be the reversal of ROS-mediated damage within the organelle, preventing depolarization. This prevention of ROS-induced depolarization would allow anterograde transport of mitochondria to proceed as normal, resulting in an efficient repopulation of spent mitochondria in the distal regions of the cell. In light of our recent study showing aberrant interactions between Drp1 and Aβ, it is likely that the induction of mitochondrial fission also plays a role in creating an abnormal distribution of mitochondria in Tg2576 neurons [114]. A positive effect of pharmacologically targeting Drp1 has been demonstrated in a cell model of Parkinson’s disease where mitochondrial division inhibitor-1 was shown to improve cell survival after 6-hydroxydopamine insult [115]. Furthermore, mito-Q treatment in the same model prevented Drp1 dependent fission and subsequent cell death [116]. Clarification about the mechanism by which Aβ, Drp1, and ROS interact may provide further drug targets for therapeutic intervention in AD.

## 8. Conclusions and Future Directions

Recent developments in AD research revealed that aging- and Aβ-induced mitochondrial abnormalities and synaptic degeneration are early events in AD pathogenesis. More specifically, Aβ-induced abnormal mitochondrial dynamics (increased fission and decreased fusion) is a critical factor that is responsible for mitochondrial structural and functional changes in AD neurons. Research utilizing primary neuronal cultures from AβPP transgenic mice also revealed defective axonal transport of mitochondria, abnormal mitochondrial distribution, reduced functionally active mitochondria at synapses, and oligomeric Aβ accumulation at synapses. These findings further suggest that Aβ is toxic to mitochondria and synapses in AD neurons.

There is urgent need to develop molecules that reduce Aβ-induced mitochondrial and synaptic toxicities, and also to develop molecules that remove/sequester Aβ from AD neurons. There is significant progress in the development of mitochondria-targeted molecules, including MitoQ and also cell-permeable small peptides, such as SS31. These mitochondria-targeted molecules have been tested by several laboratories, using cell and animal models of neurodegenerative diseases and preliminary reports have been positive. More specifically, SS31 has been found to be protective against Aβ-induced mitochondrial and synaptic deficiencies in AD neurons and appears to be a promising molecule to test in humans with AD.
